# Inanspruchnahme des Rettungsdiensts bei Suizidversuchen im Verlauf der SARS-CoV-2-Pandemie

**DOI:** 10.1007/s10049-022-01107-8

**Published:** 2022-12-20

**Authors:** Stefan Thate, Julia S. Volmerg, Frank Leenderts, Raphael Majeed, Linus Grabenhenrich, Rainer Röhrig, Insa Seeger

**Affiliations:** 1 Stadt Oldenburg – Feuerwehr, 26105 Oldenburg, Deutschland; 2 Oldenburger Forschungsnetzwerk Notfall und Intensivmedizin (OFNI), Carl von Ossietzky Universität, Oldenburg, Deutschland; 3 Institut für Medizinische Informatik, Medizinische Fakultät, RWTH Aachen, Aachen, Deutschland; 4 Großleitstelle Oldenburger Land AöR, Oldenburg, Deutschland; 5 Methodenentwicklung und Forschungsinfrastruktur, Robert Koch-Institut, Berlin, Deutschland; 6 Charité – Universitätsmedizin, Berlin, Deutschland

**Keywords:** Notfallmedizin, Notfallrettung, Rettungsleitstelle, Selbstmord, COVID-19, Emergency treatment, Emergency medical services, Emergency medical dispatch, Suicide, COVID-19

## Abstract

**Hintergrund:**

Die Pandemie hat zu Veränderungen in der Notfallversorgung mit atypischen Schwankungen der Einsatzzahlen geführt. Dies wurde u. a. mit dem veränderten Verhalten und einem gesteigerten Belastungsempfinden der Bevölkerung erklärt. Bestehende Untersuchungen geben Hinweise auf das gesteigerte Auftreten von psychischen Krankheitsbildern in der Notfallversorgung bei fortwährender Pandemie.

**Ziel:**

In diesem Beitrag wird ein Zusammenhang zwischen der COVID-19-Pandemie und dem Auftreten von Einsatzstichworten im Kontext Suizid in sechs unterschiedlich strukturierten Rettungsdienstbereichen untersucht.

**Methodik:**

Es handelt sich um eine retrospektive Querschnittstudie basierend auf der Routinedokumentation einer integrierten Leitstelle mit deskriptiver und explorativer Datenanalyse. Die Daten werden anhand siedlungsstruktureller Kreistypen aufgeschlüsselt und mit dem vom Robert-Koch-Institut (RKI) dokumentierten Wert der COVID-19-Krankheitsfälle der letzten 7 Tage/100.000 Einwohner und Pandemiephasen ins Verhältnis gesetzt.

**Ergebnisse:**

Es zeigt sich in Phase 1 und 2a ein Absinken des Einsatzaufkommens während der Pandemie. Zudem stellt sich eine Verschiebung der Dispositionsfälle mit Suizidkontext nach Strukturtypen in Phase 3 dar. Einer gesunkenen Einsatzrate im dünn besiedelten ländlichen Kreis steht eine Steigerung in der Großstadt gegenüber. Die Umstellung des Leitstellensystems zum 16.03.2021 zeigt eine deutliche Steigerung der Einsatzstichworte im Kontext Suizid.

**Diskussion:**

Die Ausprägung der Resilienz der Landbevölkerung erscheint in Phase 3 stärker ausgeprägt. Eine kontinuierliche „mental health surveillance“ unter Einbeziehung auch rettungsdienstlich erhobener Daten kann wertvolle Erkenntnisse liefern. Die Studie zeigt zudem die Notwendigkeit einer Standardisierung von Leitstellendaten auf.

## Abstract (en)

**Background:**

The pandemic has caused several changes in the emergency care system. The deployment figures in emergency medical services have shown atypical fluctuations. This has also been explained by changes in behavior and an increased sense of stress among the population. Existing research provides hints for the increased incidence of mental health symptoms in emergency care during ongoing pandemics.

**Objective:**

In this context, this paper examines the occurrence of emergency medical services calls related to the keyword suicide in relation to total calls.

**Methods:**

This is a retrospective cross-sectional study based on routine documentation from a fire and rescue dispatch center with descriptive and exploratory data analyses. The data are divided by settlement-structural county types and compared with incidences and pandemic phases.

**Results:**

Phase 1 and 2a show a decrease in the number of dispatches during the pandemic. In addition, there is a shift in the number of dispatch cases with a context of suicide by structure types in phase 3. A decreased dispatch rate in the sparsely populated rural county is offset by an increase in the metropolitan area. Changes made to the control center system resulted in an increase in the number of dispatch cases in the context of suicide.

**Conclusion:**

Continuous mental health surveillance, including data collected by emergency medical services, can provide valuable insight. The study also highlights the need for standardization of emergency dispatch center data.

## Hintergrund

Während der ersten COVID-19-Welle im Frühjahr 2020 wurde eine Reduktion um bis zu 38 % der Vorstellungen in deutschen Notaufnahmen verzeichnet [21]. Dies stand im Gegensatz zu den in den Vorjahren beobachteten steigenden Fallzahlen klinischer Notfallversorgung, welche auch im Rettungsdienst registriert wurden [3, 17]. Als Ursache für den Rückgang vermuten Ramshorn-Zimmer et al. die Maßnahmen zur Kontaktreduzierung [6] sowie Ängste und Befürchtungen der Bevölkerung vor einer Infektion [15]. Eine von Tschaikowsky et al. durchgeführte Studie zeigte im Vergleich Februar bis April 2019 zu 2020 eine Zunahme der Beschwerdebilder „auffälliges Verhalten“ und „psychiatrische Erkrankung“ zur Gesamtfallzahl auf [26]. Pirkis et al. untersuchten generelle psychische Effekte und prüften Einflüsse auf die Suizidrate in der ersten Welle der COVID-Pandemie und konnten keine Zunahme von Suizidfällen feststellen [12]. Dass sich die psychische Gesundheit mit zunehmender Pandemiedauer verändert, scheint wahrscheinlich. Matsumoto et al. haben den Zusammenhang zwischen Pandemie und psychischer Gesundheit untersucht und kamen zu dem Schluss, dass neben den Gesundheitsrisiken auch die eingeführten sozialen Beschränkungen zu einer Herabsetzung des sozioökonomischen und soziopsychologischen Status geführt haben [9].

Das RKI definierte eine retrospektive Phaseneinteilung zur Beschreibung des COVID-19-Geschehens in Deutschland 2020/2021 (RKI-Phasen) (Tab. 1; [25]). In einer ersten Untersuchung von Einsatzschwerpunkten in der Stadt Oldenburg wurde eine Steigerung der Alarmierungsstichworte von 26 (2018) bzw. 23 (2019) auf 56 (2020) im Kontext mit Suizid in der RKI-Phase 3 festgestellt [18, 23]. Ausgehend von diesem Ergebnis erfolgt mit dieser Studie eine Ausweitung der bis zu diesem Zeitpunkt betrachteten Daten über einen verlängerten Zeitraum und insgesamt sechs Rettungsdienstbereiche mit unterschiedlicher Infra- und Bevölkerungsstruktur. Der Einfluss von Bevölkerungs- und Infrastruktur im Einsatzaufkommen unter Pandemiebedingungen kann auch aus der von Flemming et al. im ersten Halbjahr 2020 dokumentierten Beobachtung der Auslastung von Intensivbetten in Niedersachsen und Bremen geschlossen werden [5].

**Table 1:** 

Zeitraum	2020/2021	Anzahl KW
Phase 1 (P1)	KW 10–20	11
Phase 2a (P2a)	KW 21–30	10
Phase 2b (P2b)	KW 30–39	9
Phase 3 (P3)	KW 40–8	22
Phase 4 (P4)	KW 9–23	15

Das Ziel dieser Studie ist, die Inanspruchnahme des Rettungsdiensts im Zusammenhang mit Einsatzstichworten im Kontext Suizid während der Pandemie zu untersuchen. Folgende Fragestellungen sollen beantwortet werden: Kam es im untersuchten Verlauf der Pandemie zu einer Veränderung der Anzahl von Einsätzen mit dem Einsatzstichwort „Suizid“ bzw. „Suizidversuch“? Besteht ein Unterschied zwischen städtischen und ländlichen Regionen?

## Methodik

### Studiendesign

Es handelt sich um eine retrospektive Querschnittstudie basierend auf der Routinedokumentation einer für sechs Gebietskörperschaften zuständigen integrierten Leitstelle für Rettungsdienst, Krankentransport und Feuerwehr mit deskriptiver und explorativer Datenanalyse.

### Population

Initial wurden alle Notfalleinsätze im Zuständigkeitsbereich der Großleitstelle Oldenburger Land AöR (GOL) ausgewertet. Dieser umfasst insgesamt 765.374 Bürger*innen [8]. Die Aufschlüsselung der Daten wurde nach Regionen und Raumabgrenzung gemäß dem Bundesinstitut für Bau‑, Stadt- und Raumforschung (BBSR) vorgenommen. Grundlage sind die siedlungsstrukturellen Kreistypen des BBSR [1]. Die GOL ist für sechs unterschiedlich organisierte Rettungsdienstbereiche zuständig. In den Städten Delmenhorst und Oldenburg wird die Leistungserbringung durch Berufsfeuerwehren mit Unterstützung durch Beauftragte geleistet, während in den Landkreisen Oldenburg und Cloppenburg ausschließlich Hilfsorganisationen beauftragt sind. Im Landkreis Wesermarsch wird der Eigenbetrieb des Landkreises durch einen Beauftragten ergänzt und im Landkreis Ammerland gibt es eine gemeinsame GmbH aus Landkreis und Hilfsorganisationen.

### Datenerschließung/Datenmanagement

Die Daten wurden für den Zeitraum vom 01.01.2018 bis zum 09.05.2021 aus dem Leitstellensystem anonymisiert extrahiert und ausgewertet. In die Studie eingeschlossen wurden die Einsätze von allen Notfallrettungsmitteln.

Für die Studie wurden Einsatzdatum und -uhrzeit, Typ des Einsatzmittels, Einsatzstichwort, Sondersignal (ja/nein), Name des Zielorts und Einsatzzeit anonymisiert extrahiert. Aus dem Einsatzdatum wurde die Variable „Kalenderwoche“ (KW) gebildet. Es wurden alle Einsätze, die mit dem Funkstatus „3 – Einsatz übernommen“ durch das alarmierte Rettungsmittel quittiert wurden, einbezogen. Im weiteren Verlauf wurden die Einsatzstichwörter mit einem suizidbedingten Einsatzanlass ausgewertet. Ausgeschlossen wurden alle Datensätze, bei denen es sich um Testläufe, Fehleinsätze oder unvollständige Datensätze handelte. Die Daten wurden – unterteilt nach KW – deskriptiv analysiert. Es wurde die wöchentliche, absolute und pro 100.000 Einwohner relative Häufigkeit von Rettungsdiensteinsätzen mit dem Einsatzstichwort „Suizid“ bzw. „Suizidversuch“ ausgewertet. Teile der Daten wurden bereits für einen Konferenzbeitrag verwendet [23].

Im Einsatz ist das Einsatzleitsystem Siveillance Command MP4.15 (Siemens AG, RC-DE BT NORD, Bremen, Deutschland). Ergänzt wurde es bis zum 15.03.2021 durch das System zur strukturierten Notrufabfrage (SNA; ISE Informatikgesellschaft für Software-Entwicklung mbH, Aachen, Deutschland). Seit dem 16.03.2021 wird stattdessen als Abfrage- und Entscheidungsunterstützungssoftware ProQA Emergency Dispatch (Priority Dispatch Corp., Salt Lake City, UT, USA; mit dem Protokoll Medical Priority Dispatch System [MPDS]) eingesetzt. Mit der Qualitätsbeauftragten der GOL wurde abgeglichen, ob das jeweilige Einsatzstichwort/der Einsatzcode inhaltlich übereinstimmte und dem vorherigen Einsatzstichwort „Suizid“ entspricht. Es wurde eine Zuordnung der Daten analog der retrospektiven Phaseneinteilung des RKI vorgenommen [25]. Um in den Grafiken die Pandemiephasen nach RKI zu ergänzen, wurde Inkscape (Inkscape-Project, Software Freedom Conservancy, Inc., Brooklyn, NY, USA) [7], Version 1.1.1, genutzt.

Die Datenanalyse wurde mit R (R Project, Free Software Foundation, Boston, MA, USA) [14], Version 3.6.1, in der Entwicklungsumgebung RStudio (posit, Boston, MA, USA) [16], Version 1.1.456, in einer Doppelprogrammierung durch zwei voneinander unabhängige Medizininformatiker*innen durchgeführt. Zur Analyse wurden neben base R die Pakete zoo [29], Version 1.8–9, für die Berechnung des „rolling average“ über 5 KW und ggplot2 [27], Version 3.3.3, zur Visualisierung der Ergebnisse eingesetzt.

## Ergebnisse

Um zufällige Artefakte des Vorjahrs auszuschließen, wurden zum Vergleich des Einsatzaufkommens in der Pandemie die Jahre 2018 und 2019 gegenübergestellt. Während im Jahr 2018 in den ländlichen Kreisen zwischen 13 und 24 Einsätze je 100.000 Einwohner (Einw.) im Kontext Suizid geleistet wurden, ist der Anteil in der Großstadt Oldenburg mit 31 Einsätzen je 100.000 Einw. höher. Die Einsatzhäufigkeit im großstädtischen Bereich im Verhältnis zur Einwohnerzahl liegt auch in den nachfolgenden Zeiträumen (2019 *n* = 36, 2020 *n* = 41, 2021 *n* = 87) oberhalb der anderen Kreistypen (Tab. 2).

**Table 2:** 

		2018		2019		2020		2021 (bis 09.05.2021)	
Stadt/Landkreis	Kreistyp	Einwohner^b^	Einsätze im Kontext Suizid^a^	Einwohner^b^	Einsätze im Kontext Suizid^a^	Einwohner^b^	Einsätze im Kontext Suizid^a^	Einwohner^b,c^	Einsätze im Kontext Suizid^a^
LK Ammerland	Städtischer Kreis	124.071	22 (17,732)	124.859	21 (16,819)	125.643	14 (11,143)	125.643	42 (33,428)
LK Cloppenburg	Ländlicher Kreis mit Verdichtungsansätzen	169.348	22 (12,991)	170.682	26 (15,233)	172.632	36 (20,854)	172.632	44 (25,488)
LK Oldenburg	Ländlicher Kreis mit Verdichtungsansätzen	130.144	24 (18,441)	130.890	18 (13,752)	131.467	28 (21,298)	131.467	30 (22,819)
LK Wesermarsch	Dünn besiedelter ländlicher Kreis	88.624	13 (14,669)	88.583	23 (25,964)	88.524	13 (14,685)	88.524	32 (36,148)
Stadt Delmenhorst	Ländlicher Kreis mit Verdichtungsansätzen	77.607	14 (18,040)	77.559	18 (23,208)	77.503	13 (16,774)	77.503	22 (28,386)
Stadt Oldenburg	Kreisfreie Großstadt	168.210	31 (18,429)	169.077	36 (21,292)	169.605	41 (24,174)	169.605	87 (51,296)
*GOL (Gesamt)*	–	758.004	126 (16,622)	761.650	142 (18,644)	765.374	145 (18,945)	765.374	257 (33,578)

^a^Zahlen in absoluten Einsätzen und () relativ zu 100.000 Einw.

^b^Landesamt für Statistik Niedersachsen (2021) Bevölkerung am 31.12. (Gebietsstand: 01.01.2020). LSN-Online: Tabelle T0901001. https://www.statistik.niedersachsen.de/startseite/datenangebote/lsn_online_datenbank/. Zugegriffen: 14. November 2021

^c^Einwohnerzahlen aus dem Jahr 2020, da keine aktuellen Zahlen vorliegen

**Figure 1:**
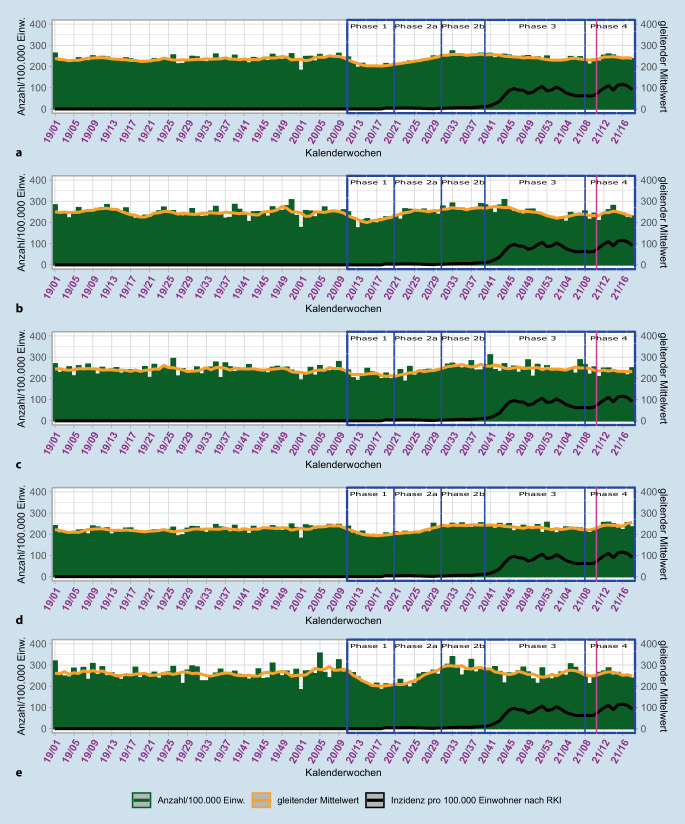

In der RKI-Phase 1 ist ein Absinken des Gesamteinsatzaufkommens in der Notfallrettung sichtbar. Das niedrigste Aufkommen zeigt sich in der KW 13/2020 bei 195/100.000 Einw. Dem folgte in der Phase 2a ein Anstieg, welcher in Phase 2b in der Spitze 273/100.000 Einw. erreichte. In Phase 3 folgte mit ansteigender Inzidenz erneut ein Absinken auf das Vorjahresniveau (Abb. 1).

**Figure 2:**
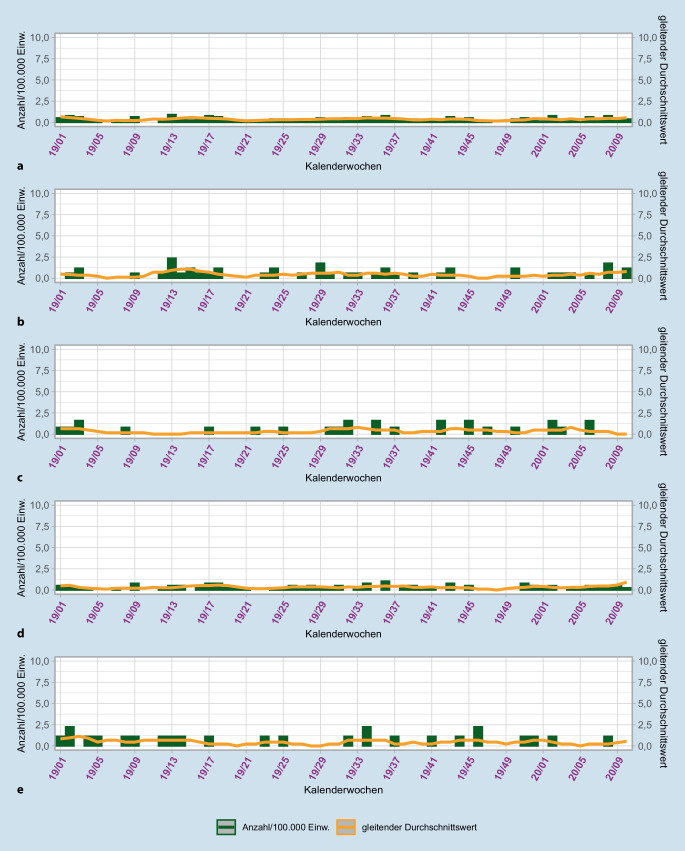

Die Daten der Einsätze im Kontext Suizid zeigen vor der Pandemie nahezu kontinuierlich einen gleitenden Durchschnitt von unter 1 (Abb. 2). In der KW 32/2018 wird einmalig ein Höchstwert von 1,05/100.000 Einw. für den gesamten Einsatzbereich ermittelt.

**Figure 3:**
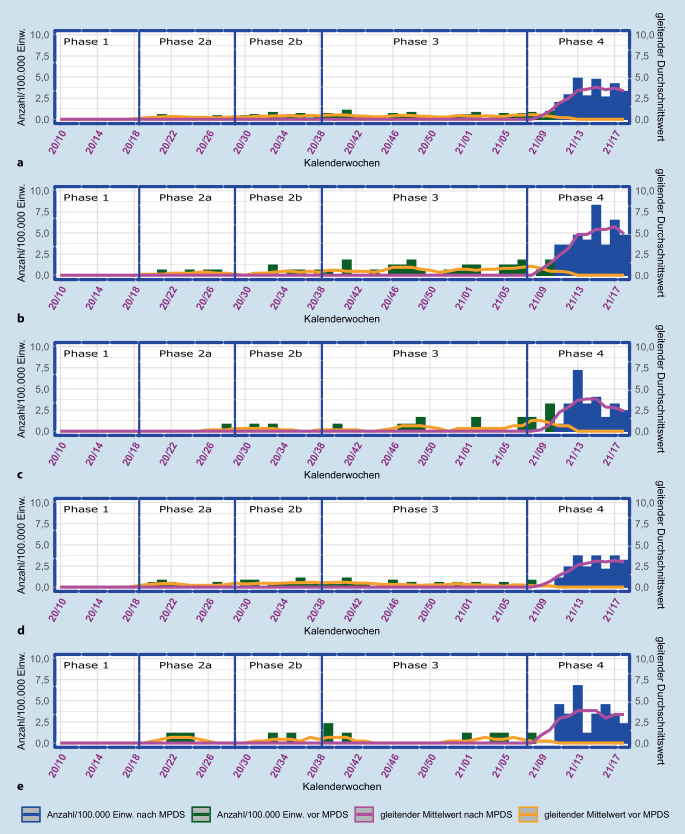

Die während der Pandemie erfassten Einsätze im Kontext Suizid zeigen bis zur Einführung der Notrufabfrage MPDS in der Phase 4 einen gleitenden Durchschnitt in der Regel unter 1. In Phase 3 wird der höchste Wert in der KW 41/2020 mit 1,05/100.000 Einw. festgestellt (Abb. 3).

Eine Betrachtung der Fälle mit Suizidkontext aus Phase 3 anhand der Strukturtypen weist Unterschiede auf. Die relative Einsatzhäufigkeit im dünn besiedelten ländlichen Kreis liegt bei 5,6 (*n* = 5) pro 100.000 Einw. In der Großstadt weist sie 15,9 (*n* = 27) auf. Die Häufigkeiten liegen bei 8,8 (*n* = 11) im städtischen Kreis und 7,3 (*n* = 28) in den ländlichen Kreisen mit Verdichtungsansätzen (Tab. 3).

**Table 3:** 

**Gesamtbereich**	Einsätze	Einsatz/100.000 Einw.	Stichwort Suizid	Suizid/100.000 Einw.	Anteil Suizid/Gesamt (%)
*2019/2020*					
KW 40‑9 (22 KW)	40.143	5245	56	7,3	0,140
*2020/2021*					
KW 40‑8 (22 KW)	40.760	5326	71	9,3	0,174
**Kreisfreie Großstadt**					
*2019/2020*					
KW 40‑9 (22 KW)	9232	5443	12	7,1	0,130
*2020/2021*					
KW 40‑8 (22 KW)	9283	5473	27	15,9	0,291
**Städtischer Kreis**					
*2019/2020*					
KW 40‑9 (22 KW)	6631	5278	11	8,8	0,166
*2020/2021*					
KW 40‑8 (22 KW)	6947	5529	11	8,8	0,158
**Ländlicher Kreis mit Verdichtungsansätzen**					
*2019/2020*					
KW 40‑9 (22 KW)	19.138	5015	25	6,6	0,131
*2020/2021*					
KW 40‑8 (22 KW)	19.420	5089	28	7,3	0,144
**Dünn besiedelter ländlicher Kreis**					
*2019/2020*					
KW 40‑9 (22 KW)	5142	5809	8	9,0	0,156
*2020/2021*					
KW 40‑8 (22 KW)	5110	5772	5	5,6	0,098

*KW* Kalenderwoche

Die im März 2021 vorgenommene Umstellung des Leitstellensystems zeigt eine deutliche Steigerung der Einsatzstichworte im Kontext Suizid auf 28,74 pro 100.000 Einw. (*n* = 220, Zeitraum KW 11–19/2021), da der spezifische MPDS-Stichwortkatalog konkretere Zuordnungen ermöglicht. Mit einer Rate von 42,45 (*n* = 72) liegt der großstädtische Bereich pro 100.000 Einw. vor den anderen Strukturtypen. Gefolgt vom dünn besiedelten ländlichen Kreis mit 31,63 (*n* = 28), städtischen Kreis mit 25,47 (*n* = 32) und ländlichen Kreis mit Verdichtungsansätzen mit 23,06 (*n* = 88). Aufgrund der unterschiedlichen Spezifität der verwendeten Abfragesysteme ist ein Vergleich mit den Vorjahren nicht möglich.

## Diskussion

Die vorliegenden Ergebnisse zeigen deutliche Unterschiede der Hilfeersuchen im Zusammenhang mit den Siedlungsstrukturen allgemein, wie auch im Kontext zu Suizidanlässen. Das rettungsdienstliche Einsatzgeschehen hat sich während der pandemischen Situation verändert. Ein allgemeiner Anstieg der Einsätze im Kontext Suizid konnte nicht ermittelt werden.

### Veränderung der Einsatzzahlen

Die kontinuierlich steigende Entwicklung des Einsatzaufkommens in der Notfallrettung wurde durch die Pandemie beeinflusst [4]. Das in der RKI-Phase 1 beobachtete Absinken des Einsatzaufkommens im Rettungsdienst [23], welches auch in den Notaufnahmen beobachtet wurde [11], fand ebenso im Oldenburger Land statt. Über den Gesamtjahreszeitraum 2020 wurde die zeitweilige Absenkung ausgeglichen und es wurden insgesamt 95.807 Rettungsdiensteinsätze geleistet, was über dem Niveau der Vorjahre liegt (2018 *n* = 88.910, 2019 *n* = 93.180).

### Unterschiede mit Einsatzstichwort „Suizid“ bzw. „Suizidversuch“

Die Ergebnisse unserer Studie zeigen zwar Schwankungen, belegen aber keine grundsätzliche Zunahme von Suizidalarmierungen im Betrachtungszeitraum. Dies deckt sich mit der bundesweiten Statistik von Suizidtoten des Statistischen Bundesamts für das Jahr 2020 [13]. Die Einführung eines kontinuierlichen, öffentlich zugänglichen Suizidregisters wie in Japan [10] und ein permanentes Sozialmonitoring des Belastungsempfindens der Bevölkerung [28] erscheinen auch in Deutschland sinnvoll, um die soziopsychologische Situation sichtbar und einschätzbar zu machen. Für eine kontinuierliche „mental health surveillance“ kann die Einbeziehung rettungsdienstlicher Daten verknüpft mit den Daten von Notaufnahmen, kassenärztlichem Bereitschaftsdienst und anderen Versorgungseinrichtungen wertvolle Erkenntnisse liefern [19].

### Unterschiede zwischen städtischen und ländlichen Regionen

Die Verläufe zeigen Abweichungen zwischen den Gesamteinsatzzahlen und den Suizidalarmierungen der Rettungsdienstbereiche im Verhältnis zur Siedlungsstruktur. Dies könnte auf Unterschiede im Belastungsempfinden der lokalen Bevölkerung hinweisen, wie es sich bei der Betrachtung der Stichwortverteilung Suizid in der Phase 3 präsentiert. Unterteilt nach Strukturtyp wird in diesem Zeitraum eine Zunahme im großstädtischen Kontext und ein Absinken im dünn besiedelten ländlichen Kreis sichtbar. Dies deckt sich mit der von Schmiedel berichteten Überrepräsentanz von Rettungsdiensteinsätzen in großstädtischen Regionen im Verhältnis zur Einwohnerzahl [20]. Die Ausprägung der Resilienz der Landbevölkerung erscheint in Phase 3 stärker ausgeprägt. Diese These wird von der aktuellen Resilienzforschung gestützt: Sie unterscheidet verschiedene Schutz- und Risikofaktoren, welche die Adaption unterstützen bzw. das Belastungserleben verstärken. Eine sichere Nachbarschaft wird hier als Schutzfaktor beschrieben, während eine große Familie auf engem Wohnraum als Risikofaktor gewertet wird [24].

### Änderung des Katalogs an Einsatzstichwörtern

In unserer Studie sollten die Fragestellungen zusätzlich auch mit Daten der Rettungsleitstelle in Aachen untersucht werden, dies war aber aufgrund der dort abweichenden Stichwortsystematik nicht möglich. Nach unserer Recherche ist die Einführung einer standardisierten und strukturierten Notrufabfrage bundesweit unterschiedlich weit fortgeschritten und das verwendete System von örtlichen Stakeholdern abhängig. Dies führt zu einem breiten Spektrum an Abfragestrategien und davon abhängigen Datensystematiken. Ohne eine Normierung der Systeme lassen sich vergleichende Untersuchungen anhand der Einsatzstichworte nur sehr limitiert vornehmen und bergen die Gefahr systematischer Fehler. Es behindert zudem ein bundesweites Benchmarking. Das Sichtbarwerden weiterer Einsatzstichworte im Suizidkontext in den Daten der GOL, welches direkt nach der Einführung eines international referenzierten Notrufabfragesystems auftrat, zeigt die Notwendigkeit einer mindestens bundesdeutschen, besser noch europäischen Einheitlichkeit und Standardisierung auf. Bereits 2018 wurde angeregt, einen Konsens über gemeinsame Standards für die Dokumentation herbeizuführen, um anschließend Einigkeit über den Grad der Genauigkeit medizinischer Dispatching-Systeme zu erreichen [2]. Die deutliche Steigerung der dokumentierten Einsatzstichworte im Suizidkontext trat unmittelbar mit ihrer Präzisierung auf. Als Ursache kommen sowohl eine Unterschätzung vor wie auch eine Überinterpretation nach Einführung infrage. Welche Auswirkungen die Präzisierung auf das Verhalten in Leitstelle und Rettungsdienst hat, sollte Gegenstand nachfolgender Untersuchungen sein. Hier wäre sowohl positives als auch negatives Priming denkbar.

### Methodendiskussion/Limitationen

Die Einführung der neuen Pilotressourcen „Gemeindenotfallsanitäter“ zum 02.01.2019 und Notfall-Krankenwagen zum 01.07.2020 kann das Dispositionsverhalten beeinflusst haben. Im laufenden Jahr 2018 wurden technische und organisatorische Anpassungen in der Dispositionssystematik vorgenommen, welche einen Vergleich der Sonderrechtseinsätze mit den Folgejahren nicht zulassen. Der Betrachtungszeitraum ab dem 16.03.2021 fällt in die Übergangsphase der Abfrageumstellung auf MPDS, hierdurch sind Unschärfen möglich. Während der Umstellung war auch die Nutzung der vorherigen Stichworte optional möglich. Für die Beantwortung der Fragestellung „Kam es im untersuchten Verlauf der Pandemie zu einer Veränderung der Anzahl von Einsätzen mit dem Einsatzstichwort ‚Suizid‘ bzw. ‚Suizidversuch‘?“ können ab diesem Zeitpunkt aufgrund nicht vorhandener Vergleichsdaten somit keine Aussagen getroffen werden.

Da im Vorfeld das Stichwort „Intoxikation“ ohne Suffixe oder Spezifikation verwendet wurde und somit nicht in die Auswertung einfließt, ist eine Steigerung der Anzahl von Suizidstichworten ab dem 16.03.2021 durch das MPDS-Protokoll 23 mit Suffix I, G, oder H möglich. Die detaillierte Übersetzung der Codes der Fa. Priority Dispatch ist rechtlich geschützt und unterliegt einem Sperrvermerk.

Die verwendeten Daten können nur Aussagen über die Einschätzung zum Dispositionszeitpunkt geben, da in der vorliegenden Systemarchitektur nicht die Daten der Einsatzdokumentation oder der Notaufnahmen verglichen werden können. Auch liegen keine adaptierbaren Daten zu gesicherten Suiziden vor.

## Ausblick

Recherchen im Rahmen dieser Studie bzgl. belastbarer lokaler Datenquellen aus Gesundheitsämtern waren ebenso erfolglos wie die Vergleichbarkeit mit Leitstellendaten der Stadt Aachen, da keine nationale Standardisierung von Daten der Notfallversorgung vorliegt. Unterschiedliche Verhaltensweisen der Bevölkerung nach Siedlungsstrukturen sollten für zukünftige, trägerübergreifende Bedarfsplanungen stärker berücksichtigt werden. Die bestehende erhebliche Inhomogenität der nationalen rettungsdienstlichen Versorgungsdichte zeigt die bisher fehlende Standardisierung bei der Bedarfsermittlung [11]. Die Einbeziehung der BBSR-Kriterien auf Ebene der Kreise und Kreisregionen bei Studien in der Notfallversorgung ermöglicht eine bessere Adaption an die tatsächlichen Bedarfe der jeweiligen Bevölkerung. Besonders im Rahmen der bevorstehenden Veränderungen in der Struktur der Notfallversorgung [17] ist ihre generelle Einbeziehung in der Untersuchung von rettungsdienstlichen Daten ein relevantes Kriterium. Optimalerweise werden solche Untersuchungen verbunden mit bundesweit einheitlich definierten Qualitätskriterien und öffentlich zugänglichen Leistungsdaten, wie beispielsweise in Baden-Württemberg [22].

## Fazit für die Praxis

- Interoperabilität und eine durchgängige Datenstruktur zu Forschungszwecken in allen Sektoren der Notfallversorgung sind dringend geboten.
- Das Erschließen von Synergien zur Verbesserung der Ergebnis- und Prozessqualität der Leistungserbringer in der Notfallversorgung erfordert die Untersuchung der vollständigen Prozesskette.
- Die Einbeziehung rettungsdienstlicher Daten bietet einen Erkenntnisgewinn zur Erforschung sozialer und gesellschaftlicher Prozesse.
- Das erhöhte Aufkommen „Einsätze im Kontext Suizid“ im städtischen Bereich zieht einen gesteigerten Präventionsbedarf nach sich. Die direkte Vernetzung eines entsprechend sensibilisierten Rettungsdiensts mit aufsuchenden Sozialdiensten sollte ausgebaut werden.
